# Effects of Optogenetic Suppression of Cortical Input on Primate Thalamic Neuronal Activity during Goal-Directed Behavior

**DOI:** 10.1523/ENEURO.0511-20.2021

**Published:** 2021-03-19

**Authors:** Tomoki W. Suzuki, Ken-Ichi Inoue, Masahiko Takada, Masaki Tanaka

**Affiliations:** 1 Department of Physiology, Hokkaido University School of Medicine, Sapporo 060-8638, Japan; 2 Laboratory of Neural Systems, The Rockefeller University, New York, New York 10065; 3 Systems Neuroscience Section, Primate Research Institute, Kyoto University, Inuyama 484-8506, Japan

**Keywords:** corticothalamic terminals, eye movements, halorhodopsin, monkey, oculomotor thalamus, single neuron activity

## Abstract

The motor thalamus relays signals from subcortical structures to the motor cortical areas. Previous studies in songbirds and rodents suggest that cortical feedback inputs crucially contribute to the generation of movement-related activity in the motor thalamus. In primates, however, it remains uncertain whether the corticothalamic projections may play a role in shaping neuronal activity in the motor thalamus. Here, using an optogenetic inactivation technique with the viral vector system expressing halorhodopsin, we investigated the role of cortical input in modulating thalamic neuronal activity during goal-directed behavior. In particular, we assessed whether the suppression of signals originating from the supplementary eye field at the corticothalamic terminals could change the task-related neuronal modulation in the oculomotor thalamus in monkeys performing a self-initiated saccade task. We found that many thalamic neurons exhibited changes in their firing rates depending on saccade direction or task event, indicating that optical stimulation exerted task-specific effects on neuronal activity beyond the global changes in baseline activity. These results suggest that the corticothalamic projections might be actively involved in the signal processing necessary for goal-directed behavior. However, we also found that some thalamic neurons exhibited overall, non-task-specific changes in the firing rate during optical stimulation, even in control animals without vector injections. The stimulation effects in these animals started with longer latency, implying a possible thermal effect on neuronal activity. Thus, our results not only reveal the importance of direct cortical input in neuronal activity in the primate motor thalamus, but also provide useful information for future optogenetic studies.

## Significance Statement

Although previous studies in songbirds and rodents have shown that corticothalamic inputs are essential for generating movement-related activity in the motor thalamus, their role in primates remains largely unknown. Here, we attempted to optogenetically suppress the corticothalamic terminals during neuronal recording from the oculomotor thalamus in monkeys performing a saccade task. We found that optical stimulation resulted in task-specific changes in the firing rate, indicating that the corticothalamic projections are engaged in neural computations for goal-directed behavior. We also observed non-task-specific changes in baseline activity that might be caused by local heating of surrounding tissue, which underscores the importance of control experiments in animals without opsin expression.

## Introduction

Numerous studies have demonstrated that a variety of neuronal signals in the motor thalamus play roles in the planning and execution of movements (Strick, 1976; Anderson and Turner, 1991; Nambu et al., 1991; Kurata, 2005). Clinically, large lesions in the motor thalamus cause hemiplegia, while a small localized inactivation in experimental animals results in more specific behavioral deficits depending on the site of inactivation (van Donkelaar et al., 2000). It is generally believed that the primary role of the motor thalamus is simply to relay signals derived from the basal ganglia, the cerebellum, and the brainstem to the motor cortical areas (Asanuma et al., 1983). However, a previous study in songbirds showed that movement-related neuronal firing in the motor thalamus largely depends on the inputs from the motor cortical areas (Goldberg and Fee, 2012). More recently, Guo et al. (2017) reported in rodents that the signals from the motor cortex are crucial for the maintenance of preparatory activity and the generation of direction-selective movement signals in the motor thalamus.

Despite such accumulating evidence, little is known about the role of direct cortical inputs in the generation of neuronal activity in the primate motor thalamus. Inactivation in the output node of the basal ganglia (i.e., the internal segment of the globus pallidus) has been shown to change the baseline firing of thalamic neurons, but it exerts only a minor effect on movement-related activity (Inase et al., 1996), suggesting that the corticothalamic projections might instead contribute to the generation of movement-related neuronal activity. On the other hand, a recent study has demonstrated that optical stimulation of corticothalamic terminals induces both excitatory and inhibitory responses in thalamic neurons at relatively long latency, indicating that the cortical inputs provide a modulatory role rather than a fast excitatory drive (Galvan et al., 2016). However, it remains unknown whether the corticothalamic projections are essential for the computation of signals relevant to volitional actions beyond the global facilitation or suppression of baseline activities in the motor thalamus. For example, cortical inputs during motor preparation might modulate thalamic neuronal activity around the time of movement onset in a manner that depends on the movement direction.

Although the motor thalamus often refers to the thalamic nuclei associated with limb movements, the region medial to the motor thalamus is also known to be involved in the control of eye movements (Alexander et al., 1986; Tian and Lynch, 1997; Wyder et al., 2003; Tanibuchi and Goldman-Rakic, 2005; Tanaka and Kunimatsu, 2011). In this study, we examined the roles of direct corticothalamic projections in neuronal activity in the oculomotor thalamus while monkeys were actively engaged in a behavioral task. Specifically, we used the self-timed saccade task, because prominent firing modulation in the oculomotor thalamus during the task has been demonstrated (Tanaka, 2007b; Wang et al., 2018) and because focal pharmacological inactivation of these sites significantly changes the task performance (Tanaka, 2006). Our primary objective was to test whether optogenetic suppression of corticothalamic terminals could induce task-specific neuronal modulation. We chose the supplementary eye field (SEF) as a vector injection site, because abundant projections from the SEF to the thalamus (Huerta and Kaas, 1990; Shook et al., 1991) might be particularly important for the task, given the clear neural modulation in the SEF during the task and the effects of electrical microstimulation on self-timing (Kunimatsu and Tanaka, 2012).

## Materials and Methods

### Animal preparation and surgery

The subjects were four adult Japanese monkeys (*Macaca fuscata*, monkeys F, M, B, and Q; one female and three males; age range, 5–14 years; weight range, 6–10 kg). Two of them were also used for previous recording and behavioral experiments (monkeys F and B; Suzuki et al., 2016; Suzuki and Tanaka, 2017, 2019). All experimental protocols were evaluated and approved in advance by the Hokkaido University Animal Care and Use Comittee. In separate surgical procedures, animals were sterilely implanted with head holders, eye coils, and recording chambers under general isoflurane anesthesia. Analgesics (pentazocine and ketoprofen) were administered during and a few days after each surgery. After full recovery, the monkeys were trained on the oculomotor tasks. During the training and experimental sessions, animals sat in a primate chair in a darkened booth with their heads restrained. Horizontal and vertical eye positions were recorded using the search coil technique.

### Viral vector

Adeno-associated viral (AAV) vectors designed to express the light-driven chloride pump halorhodopsin (AAV2-CMV-eNpHR3.0-EYFP) were produced by the helper-free triple-transfection procedure and purified by affinity chromatography (GE Healthcare). The viral titer was determined by quantitative PCR using TaqMan technology (Thermo Fisher Scientific). The transfer plasmid (pAAV-CMV-eNpHR3.0-EYFP-WPRE) was constructed by inserting the eNpHR3.0-EYFP gene (kindly provided by K. Deisseroth, Stanford University) and the WPRE sequence into an AAV backbone plasmid (pAAV-CMV; Stratagene).

### Injections of adeno-associated viral vectors

**Figure 1.**
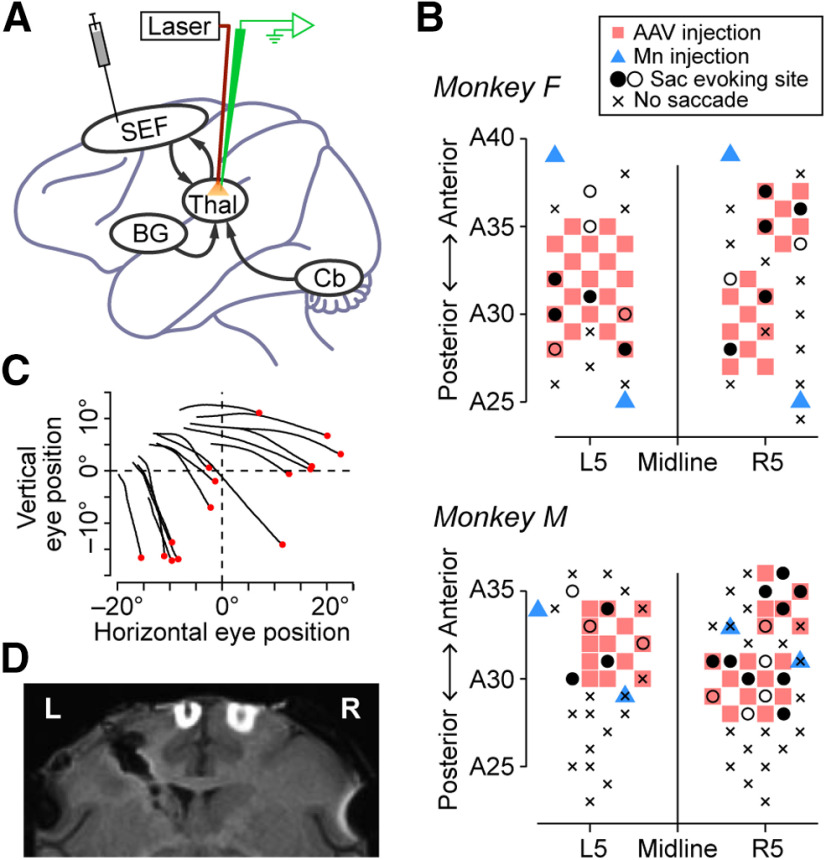
Experimental procedures and injection sites. ***A***, Schematic of the experiments. We initially injected the anterograde AAV vector (AAV2-CMV-eNpHR3.0-EYFP) into the SEF bilaterally. After the expression of halorhodopsin at the corticothalamic terminals, we recorded from single neurons in the oculomotor thalamus using optrodes with and without light delivery. BG, Basal ganglia; Cb, cerebellum; Thal, thalamus. ***B***, Stimulation and injection sites for monkeys F (top) and M (bottom). Injection sites of the viral vector and contrast agent for MRI (Mn) are shown in red squares and blue triangles, respectively. Sites evoking eye movements with different current intensities are shown separately (≤50 μA, filled circles; ≤100 μA, open circles). Sac, Saccade. Xs indicate saccade-nonevoking sites. Anteroposterior coordinates are relative to the interaural line. ***C***, Trajectories of stimulation-evoked saccades in a single session. Data during 100 ms following electrical stimulation (50 μA) are displayed, and red dots indicate eye position at the stimulation onset. ***D***, Coronal MR image of monkey F (A24). High-intensity spots indicate Mn injection sites. L, Left; R, right.

Before viral injection, the SEF was identified using electrical microstimulation (a train of 333 Hz biphasic pulses; pulse duration, 0.2 ms; stimulation duration, 100 or 150 ms; amplitude, 60–100 μA) through tungsten microelectrodes (FHC). Once eye movements were evoked by stimulation, an additional test was conducted at lower current intensity (30–50 μA; Fig. 1*B*, black circles). The vector was injected into multiple sites in the SEF of two monkeys (F and M), who received 34 and 29 μl of injection volumes, respectively (Fig. 1*B*, red squares). For each penetration, 0.5 μl of the solution was injected both at 4–6 and 2–3 mm below the thickened dura surface. Based on preliminary experiments, we assumed that the viral vector spreads ∼1.5 mm horizontally. Viral titers were 1.5 × 10^12^ genome copies (GC)/ml for both hemispheres of monkey F and 2.5 × 10^12^ and 1.1 × 10^13^ GC/ml for the left and right hemispheres of monkey M, respectively. To confirm successful injection, the magnetic resonance imaging (MRI) contrast agent [manganese chloride (Mn)] was also injected into four additional locations in each animal (Fig. 1*B*, blue triangles). Injections were performed under general isoflurane anesthesia. Optical stimulation experiments were started >1 month following viral vector injection.

### Visual stimuli and behavioral tasks

The experiments were controlled using a real-time data acquisition system (TEMPO, Reflective Computing). Visual stimuli were presented on a 27 inch liquid crystal display monitor (refresh rate, 144 Hz) located 42 cm from the eyes (visual angle, 71 × 44°). In the self-timed saccade task (see Fig. 3A), each trial began with the appearance of a fixation point (FP; white 0.6° square) at the center of the screen. When eye position remained within 3° of the FP for 400 ms, the color (and shape) of the FP changed. A blue filled square was used for monkeys B and M, but a green triangle and a cyan unfilled square were used for monkeys F and Q, respectively. After a variable delay (1200–1700 ms) following the start of fixation, a visual cue (white 0.6° square) was presented 16° right or left of the FP for 100 ms. If animals made a saccade to the remembered cue location during the 1000–1700 ms period following the cue onset, the FP was extinguished, and the monkeys received a juice reward 800 ms after the saccade. The cue reappeared immediately after saccade initiation, which was detected online when eye position deviated >4° from the FP. In many sessions, the trial was aborted if animals failed to maintain fixation on the reappeared target (cue) until reward delivery; however, even without such requirement in a minority of sessions, animals usually maintained fixation after the saccade. The data were discarded if monkeys made early saccades before the cue onset. Intertrial interval ranged from 2750 to 3950 ms. A “blank” trial (4000–4650 ms) was also randomly interleaved with saccade trials at 20% probability, in which neither visual stimuli nor rewards were presented. The blank trial was used to extend the intertrial interval (ITI).

In randomly selected half of the trials, a continuous optical stimulation (duration, 2500 ms; 1900 ms in one session) was applied to the recording sites either 100 ms before or simultaneously with the cue onset (consistent in each session). In eight sessions in monkey M, a continuous optical stimulation of 2000 ms was applied 500 ms after the cue onset. To examine the effects on spontaneous neuronal activity, optical stimulation was also delivered during half of the blank trials with the same stimulation parameter as in the self-timed saccade trials.

### Physiological procedures

We examined the effects of optical stimulation on single-neuron activities using homemade or commercially available optrodes (Doric). Homemade optrodes were made from a tungsten electrode (100 μm diameter; FHC) and optic fiber [outer diameter, 65–125 μm; numerical aperture (NA) of 0.22 or 0.37; Doric]. The electrode tip advanced 300–800 μm (usually 450–600 μm) ahead of the optical fiber. The purchased optrodes were made from a tungsten electrode (125 μm diameter) and optic fiber (outer diameter, 70 or 125 μm; NA, 0.22) with a tip distance of 200 μm. The fiber tips of the purchased optrodes and some of the custom-made ones were beveled to reduce tissue damage (Stauffer et al., 2016). The optic fiber was connected to a 589 nm laser source (589F100, Dragon Lasers), which was located outside of the experimental booth. The light power at the optrode tip was measured just before each experiment using a photodiode power sensor (PD300-1W, Ophir Photonics) and ranged from 0.7 to 5.7 mW (median, 3.7 mW; interquartile range, 2.8–3.9 mW) and 1.1–9.5 mW (median, 3.4 mW; interquartile range, 2.9–3.7 mW) for the animals with and without viral vector injection, respectively. To avoid light leakage, polyimide tubes covering the optic fibers were painted with black nail polish and a permanent marker. When we needed to connect optical fibers in the experimental booth, a metal or ceramic ferrule was carefully covered with black paper and plastic tapes. Furthermore, the recording chamber and surrounding area were constantly illuminated during the experiments with a high-luminance light-emitting diode (LED, 590 nm) placed just above the recording chamber and/or with an array of white LEDs attached to the ceiling of the experimental booth.

**Figure 2.**
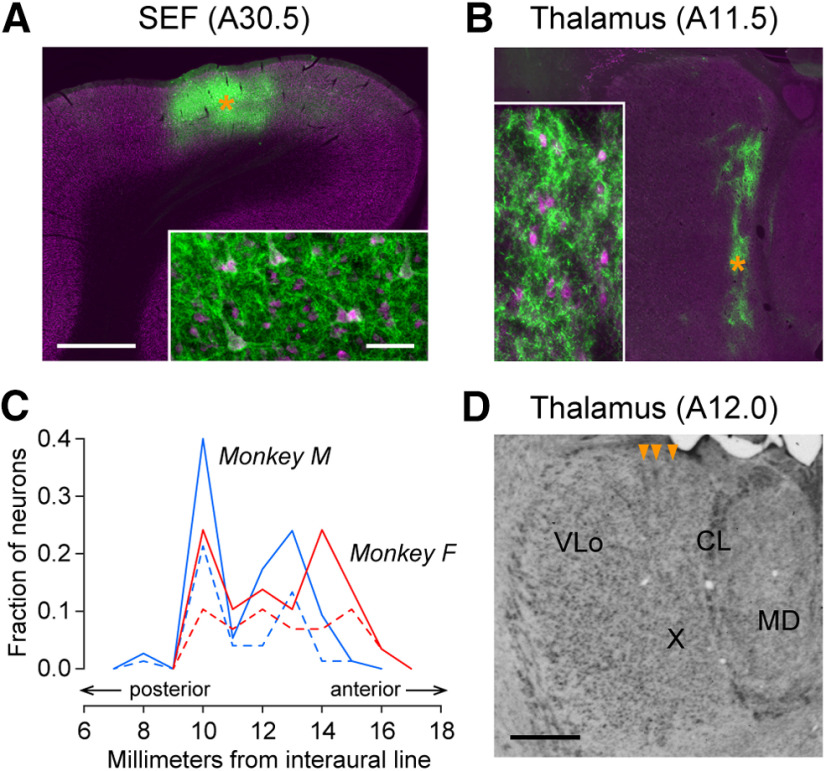
Opsin expression and recording sites. ***A***, ***B***, Fluorescent immunohistochemical staining for EYFP (green) and NeuN (magenta) in representative sections through SEF (***A***) and thalamus (***B***) in monkey F. Orange asterisks denote the locations of higher-magnification insets. Scale bars: low magnification, 2 mm; high magnification, 50 μm. The distance from the interaural line is indicated in parentheses. ***C***, Anteroposterior distribution of the recorded thalamic neurons. Different colors represent different animals. Solid and dashed lines denote the proportions of all recorded neurons (monkey F, *n *=* *29; monkey M, *n* = 75) and those exhibiting task-specific effects of optical stimulation, respectively. ***D***, Nissl staining of thalamic recording sites in monkey F. Orange arrowheads point to electrode tracks. Scale bar, 2 mm. VLo, Oral division of the VL nucleus; X, area X of Olszewski (1952).

**Figure 3.**
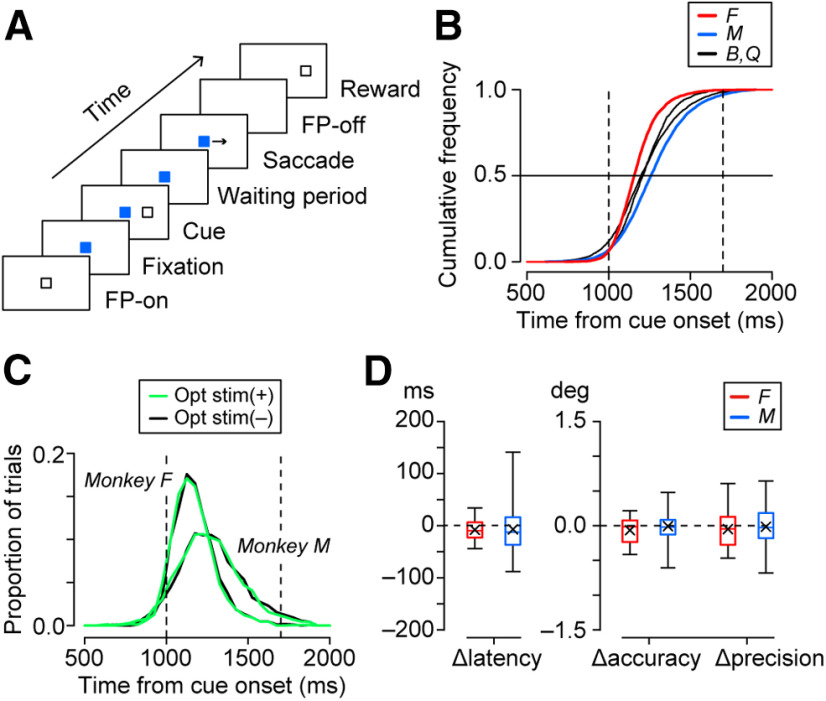
Behavioral paradigm and performance of the animals. ***A***, In the self-timed saccade task, monkeys were trained to generate a self-initiated saccade to the location of a briefly (100 ms) presented visual cue >1000 ms following the cue onset. ***B***, Cumulative distributions of saccade timing during recording sessions in all four monkeys (monkey F, *n *=* *3461 trials; monkey M, *n* = 8240 trials; monkey B, *n* = 3906 trials; monkey Q, *n* = 2124 trials). Animals obtained liquid reward for saccades generated during the time window delimited by vertical dashed lines. ***C***, Comparison of the distributions of saccade latency in trials with (green trace) and without (black) optical stimulation in the vector-injected monkeys. ***D***, Summary of the effects of optical stimulation on saccade latency, accuracy, and precision. Each box-and-whisker plot represents the median, quantiles, and range of the data. Xs indicate the means. Different colors indicate different monkeys. Accuracy and precision were quantified by measuring the mean sizes and SDs of the horizontal saccade error, respectively. The values for opposite saccade directions were averaged for each session.

Optrodes were inserted through a 23 gauge stainless steel guide tube and were advanced using a micromanipulator (MO-97S, Narishige). Signals were referenced to either the guide tube or an Ag-AgCl disk electrode placed on the dura mater, and were amplified and bandpass filtered (300 Hz–10 kHz, or 300 Hz–5 kHz in a few cases). We searched for task-related neurons while monkeys performed a task set consisting of conventional memory-guided saccade trials (Hikosaka and Wurtz, 1983) and self-timed saccade trials with three different waiting periods, where different conditions were indicated by different colors and shapes of the FP (Suzuki and Tanaka, 2019). Action potentials of a single neuron were isolated online using a spike detector with a template matching algorithm (ASD, Alpha Omega Engineering). Data from single neurons were collected during the main task block consisting of self-timed trials with one waiting interval (1000–1700 ms; see Fig. 3*A*) and blank trials. Neuronal data with less than five trials in any of the six conditions (self-timed saccade trials in the opposite directions and blank trials in the presence or absence of optical stimulation) were excluded from further analyses. In total, 104 thalamic neurons were examined in monkeys with vector injections (monkey F, *n *=* *29; monkey M, *n* = 75). In monkeys without vector injections (control animals, monkeys B and Q), 13 thalamic neurons and 32 cortical neurons were examined. Recording sites in the thalamus spanned 8−16 mm anteriorly from the interaural line and 3−6 mm laterally from the midline (Fig. 2*C*). Those in the cortex ranged from 10 to 16 mm anteriorly from the interaural line and from 3 to 6 mm laterally from the midline.

### Histological procedures

At the end of experiments in monkeys F and M, several electrolytic lesions were made by passing a direct current through a tungsten electrode (tip negative, 10–20 μA for ∼60 s, 800–1000 μC). Then, the animals were deeply anesthetized with sodium pentobarbital (60 mg/kg, i.p.) and perfused transcardially with 0.1 m PBS followed by 3.5% paraformaldehyde. The brains equilibrated with 30% sucrose in PBS were cut in the coronal plane at 50 μm thickness. Every 10th section was stained with cresyl violet, and the recording locations were reconstructed based on electrode tracks and electrolytic lesions.

To visualize the immunoreactive signals of eNpHR3.0-EYFP and NeuN, the sections were immersed in 1% skim milk for 1 h and incubated overnight with rabbit anti-green fluorescent protein antibody (1:1000 dilution; Thermo Fisher Scientific) and mouse monoclonal anti-NeuN antibody (1:1000 dilution; Millipore) in PBS containing 1% normal donkey serum. The sections were then incubated in the same fresh medium containing Alexa Fluor 488-conjugated donkey anti-rabbit IgG (1:400 dilution; Jackson ImmunoResearch) and Cy3-conjugated donkey anti-mouse IgG (1:400 dilution; Jackson ImmunoResearch). Images of the sections were digitally captured using an optical microscope equipped with a high-grade CCD camera (model BX-900, Keyence).

### Data acquisition and statistical analysis

**Figure 4.**
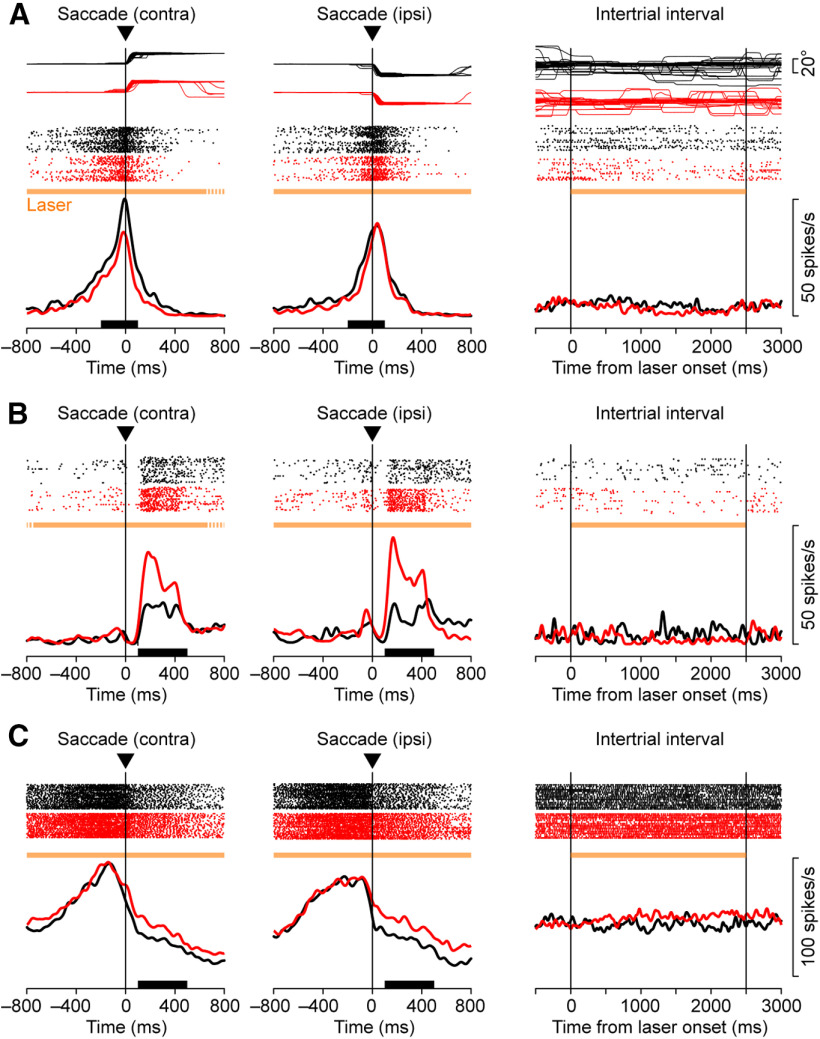
***A***, A thalamic neuron exhibiting task-specific effects of optical stimulation recorded from monkey M. Eye position traces (top), spike rasters (middle), and spike density profiles (bottom) are aligned on saccade initiation (left and middle) or the onset of laser illumination during the blank trial (right). Red and black traces represent the data in trials with and without optical stimulation, respectively. These trials were presented randomly. The horizontal orange bar in each panel indicates the period of light illumination. Black bars on the *x*-axes denote the time window for the analysis (saccade period). Note that optical stimulation clearly reduced the firing modulation for contraversive (contra) saccades, but not for ipsiversive (ipsi) saccades. ***B***, ***C***, Other examples of thalamic neurons with temporally specific laser effects recorded from monkeys M (***B***) and F (***C***). Dotted parts of horizontal orange bars denote the period where light stimulation was not applied in a fraction of trials. All three neurons were recorded from subdivisions of the VL nucleus.

**Figure 5.**
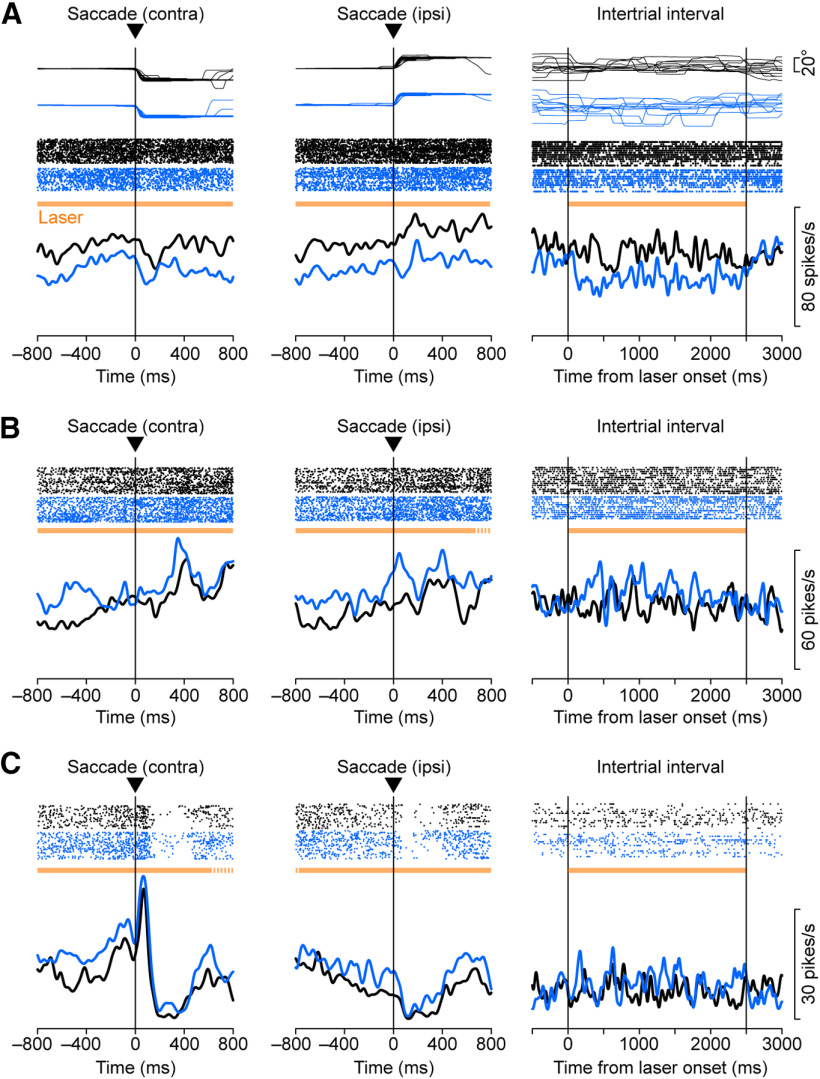
Three thalamic neurons exhibiting non-task-specific effects of optical stimulation recorded from monkeys M (***A***, ***B***) and B (***C***). Note that monkey B received no vector injection. Neurons in ***A***, ***B***, and ***C*** were recorded from the VL, VA, and VL nuclei, respectively. Same configuration as in Figure 4. ipsi, Ipsiversive; contra, contraversive.

Data on eye position and neuronal activities were digitized at 16 bit resolution and sampled at 1 kHz, and were saved in files during experiments along with event time stamps. Data were analyzed offline using MATLAB (MathWorks). Trials with goal-nondirected saccades (>8° from the cue location, 3.5%) were excluded from the analysis. Self-timed trials with saccade latencies (measured from the cue onset) <600 or >1900 ms were also removed (2.1%). To display the time courses of neuronal activity (see Figs. 4, 5), spike density functions (Gaussian kernel, σ = 20 ms) were computed from the probability of action potential occurrence every millisecond, while all quantitative measures were based on actual spike counts at specific time windows except for the analysis of population latency of stimulation effects with high temporal resolution (see below).

**Figure 6.**
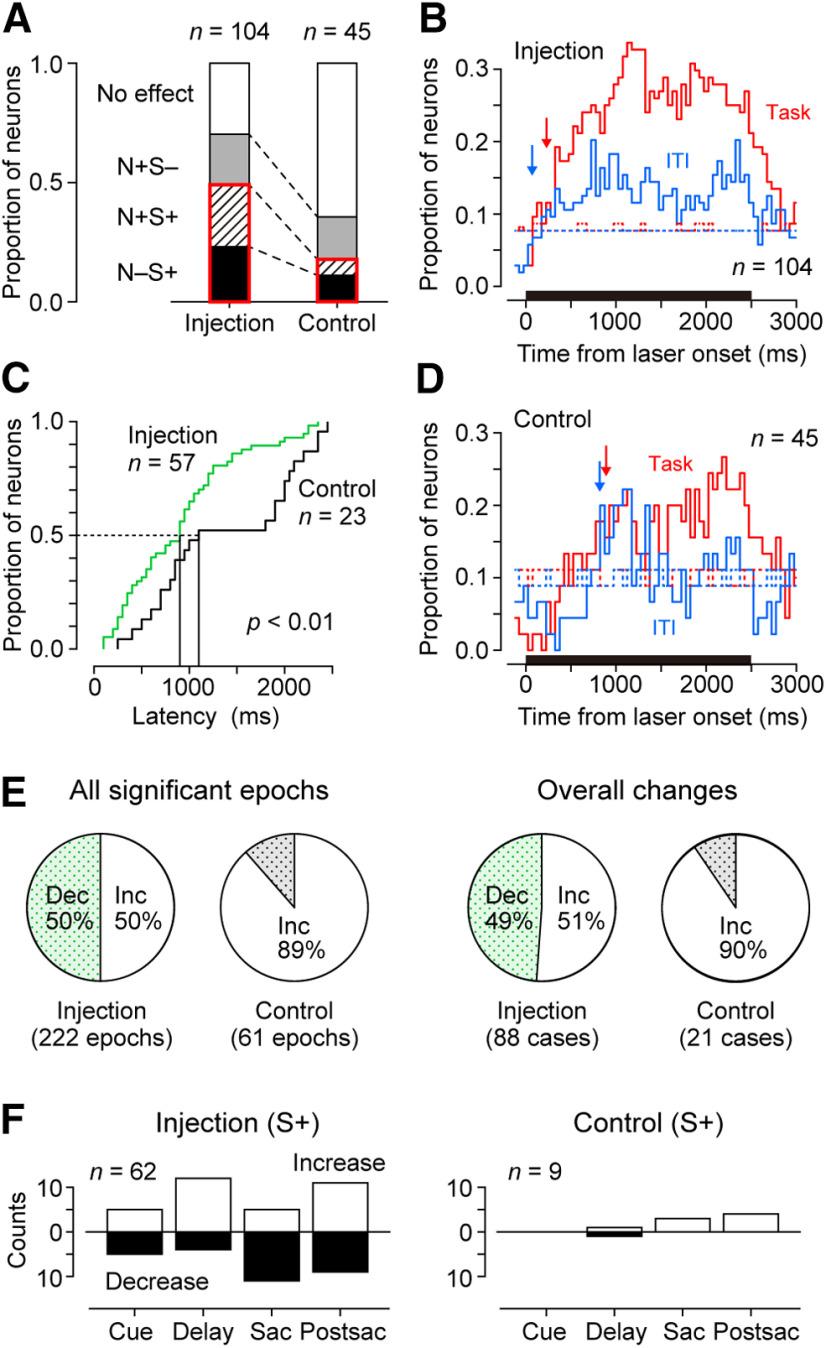
Quantitative analyses of the effects of optical stimulation. ***A***, Proportions of neurons exhibiting four different types of response in vector-injected (left) and noninjected (control, right) animals. S+/– and N+/– indicate the presence/absence of task-specific and non-task-specific changes in firing rates during optical stimulation, respectively. The proportions of neurons with task-specific changes are highlighted by red rectangles. ***B***, ***D***, Time courses of the proportions of neurons with significant laser effects for the animals with vector injections (***B***) and controls (***D***). Red and blue traces represent the data during the saccade trials (Task) and those during the blank trials (ITI), respectively. Downward arrows indicate the population latencies computed with a 10 ms resolution (see Materials and Methods). Dotted lines represent the 95th percentiles of the permuted data. ***C***, Cumulative frequency of latencies measured for individual neurons. Green and black traces denote latencies for the animals with vector injections and controls, respectively. ***E***, Proportions of cases with significant increase (Inc) and decrease (Dec) in activities during optical stimulation. Left pies summarize the data examined separately for different epochs and saccade direction. Right pies indicate the results of overall changes during either of the three conditions (right/left saccades and ITI). ***F***, Number of epochs with task-specific stimulation effects in vector-injected (left) and control (right) animals. Open and filled bars indicate facilitatory and suppressive effects, respectively. Sac, Saccade; Postsac, postsaccade.

For the quantitative analyses of optical stimulation, we initially assessed the changes in spike count over the entire stimulation period (1900, 2000, or 2500 ms). For each of the three task conditions (rightward saccades, leftward saccades, and the blank trial), the spike counts with and without optical stimulation were compared using a two-sided Wilcoxon rank-sum test with false discovery rate (FDR) correction for multiple comparisons (*p *<* *0.05/3). Neurons with a significant change in activity in any condition were considered to have non-task-specific effects (see Fig. 6*A*, N+). To evaluate the task-specific effects of light stimulation (see Fig. 6*A*, S), we measured the spike counts during the following four time epochs in each saccade condition: (1) 100–400 ms from the cue onset (cue period); (2) 200–600 ms before saccade initiation (delay period); (3) a 300 ms period starting from 200 ms before saccade initiation (saccade period); and (4) 100–500 ms following saccade initiation (postsaccade period). For each epoch, neuronal modulation during optical stimulation was quantified by computing the effect size (Cohen’s *d*), defined as (μ_w_ − μ_wo_)/sqrt[(σ_w_^2^ + σ_wo_^2^)/2], where μ and σ indicate the mean and SD of spike counts with (w) or without (wo) optical stimulation, respectively. Statistically significant modulation was determined by comparing the permuted data (10,000 iterations), where trials were randomly reassigned with and without optical stimulation while the number of trials in each condition remained unchanged. For each time epoch with significant modulation (i.e., those deviated from the middle 95% distribution of the effect size computed for the permuted data), we examined the context dependency. Specifically, to detect direction-specific modulations, we computed the difference in effect size between the saccade directions for each time epoch and each neuron. We then assessed whether the value was >97.5% or <2.5% of the corresponding value derived from the permuted data. Temporally specific modulations were designated when the effect size for only a single time epoch in a given condition was statistically significant.

To assess the latency of stimulation effects in each condition, data in trials with and without optical stimulation were compared during every 200 ms window (50 ms step) starting from stimulation onset (see Fig. 6*B–D*). Statistical significance was evaluated by two-sided Wilcoxon rank-sum test with FDR correction for the three conditions (*p *<* *0.05/3, self-timed trials in the opposite directions and the blank trial). For each task condition, the center of the first bin of the two consecutive bins with a significant difference was defined as the onset of stimulation effects, and the latency for each neuron was the earliest one across the three conditions (see Fig. 6C). To compare the population latency of stimulation effects between the two animal groups (see Fig. 6*B*,*D*), the significant difference for each bin was evaluated by two-sided Wilcoxon rank-sum test with FDR correction for saccade directions (Task, *p *<* *0.05/2) or the blank trial (ITI, *p *<* *0.05). The first time point when four consecutive bins exceeded the 95th percentiles of the permuted data were detected for each group. Then, spike density functions (Gaussian kernel, σ = 20 ms) were obtained for ±300 ms periods around these time points, and population latencies were determined at 10 ms resolution as the first time when the proportion exceeded the 95th percentiles of the permuted data for >50 ms (see Fig. 6*B*,*D*, downward arrows).

## Results

### Injection sites of the viral vector and halorhodopsin expression

To express halorhodopsin in the corticothalamic terminals, we injected an adeno-associated viral vector (AAV2-CMV-eNpHR3.0-EYFP) into the SEF of both hemispheres in two monkeys (F and M; Fig. 1*A*). We targeted the SEF because previous reports demonstrated that prominent neural activities associated with self-initiated saccades and electrical stimulation changed behavioral performance (Kunimatsu and Tanaka, 2012). Before the vector injections, the SEF was identified by electrical microstimulation (see Materials and Methods). Figure 1*C* displays the trajectories of evoked eye movements toward the contralateral upper visual field at an example stimulation site. The median latencies of evoked saccades averaged 75.5 ± 31.7 ms (SD, *n *=* *35 sites; range, 33–163 ms), which was in good agreement with previous studies (Schlag and Schlag-Rey, 1987; Russo and Bruce, 1993; Park et al., 2006). Figure 1*B* shows the injection sites of the viral vector (red squares) and Mn (blue triangles). Successful injection was confirmed by the high-intensity spots at the Mn injection sites on MR images (Fig. 1*D*).

We performed physiological experiments in the oculomotor thalamus later than 1 month following the vector injections. The recording sites extended from 8 to 16 mm anterior to the interaural line and from 3 to 6 mm from the midline. Figure 2*C* summarizes the distributions of all recorded neurons (solid lines, *n *=* *104) and the neurons showing the task-specific effects of optical stimulation (dashed lines, *n *=* *51, see below) along the anteroposterior axis of each monkey. These thalamic neurons were located in the ventroanterior (VA; *n *=* *38), ventrolateral (VL; *n *=* *45), and mediodorsal (MD; *n *=* *21) nuclei, while those recorded from the centrolateral (CL) nucleus were included in either the MD or the VL group. The proportions of neurons with task-specific stimulation effects were not different across the VA, VL, and MD nuclei (χ^2^ test: χ^2^ = 0.70, *p *=* *0.71). After completion of the experiments, we obtained histological sections (Materials and Methods) and verified the halorhodopsin expression and recording locations (Fig. 2*A*,*B*,*D*). We found patches of labeled corticothalamic terminals rostrally in the dorsomedial aspect of the oral division of the ventrolateral nucleus, including area X of Olszewski (1952), and caudally in the dorsolateral aspect of the mediodorsal nucleus as well as in the CL nucleus (Fig. 2*B*), which largely overlapped our recording sites (Fig. 2*D*).

### Lack of behavioral changes during optical stimulation

In the self-timed saccade task (Fig. 3*A*), monkeys obtained rewards for saccades generated within 1000–1700 ms following the visual cue. Figure 3*B* displays the cumulative relative distributions of saccade latencies, showing that all monkeys were able to measure elapsed time and generate self-initiated saccades in appropriate timing. We recorded from 104 thalamic neurons using optrodes in the animals with the vector injections (monkeys F and M). As control experiments, we also recorded from 13 thalamic and 32 overlying cortical neurons in monkeys without the vector injection (monkeys B and Q) using the same procedures. In all four animals, optical stimulation (2500 ms) was conducted in a random 50% of trials, which started 100 ms before or simultaneously with the time of the cue onset (see Materials and Methods).

We first compared saccade parameters between trials with and without laser stimulation to rule out the possibility that any change in neuronal activity resulted from the light-induced changes in behavioral performance. While distributions of saccade latencies differed among monkeys (Fig. 3*B*), those for the two conditions in each monkey were very similar and statistically indistinguishable for both the vector-injected (Kolmogorov–Smirnov test, *p *>* *0.17; Fig. 3*C*) and the control groups (*p *>* *0.43). Furthermore, saccade latencies statistically differed between the stimulation conditions in only 3 of 104 sessions (2.9%; two-sided Wilcoxon rank-sum test, *p *<* *0.025 for each saccade direction) in monkeys with the vector injections and in none of the 45 sessions in the control animals. We also examined the accuracy and precision of self-timed saccades by computing the mean sizes and SDs of the horizontal error, respectively. The box-and-whisker plots in Figure 3*D* summarize the changes in the three saccade parameters during optical stimulation in animals with the vector injections; these changes were not statistically different from 0 (one-sample *t* test, *p *>* *0.05). This was also true in the control animals (*p *>* *0.05, data not shown). Therefore, we concluded that our optical stimulation modulated only a small number of neurons and did not affect behavioral performance.

### Light-induced changes in neuronal activity

Figure 4*A* illustrates an example VL thalamic neuron exhibiting a burst of activity associated with saccades. In the absence of optical stimulation (Fig. 4*A*, black traces), the activity was directional and the neuronal firing rate for rightward (contraversive) saccades (Fig. 4*A*, left; measured at black bar) was greater than that for leftward saccades (Fig. 4*A*, middle; two-sided Wilcoxon rank-sum test, *p *<* *0.01). During laser stimulation (Fig. 4, horizontal orange bar), the activity for rightward saccades clearly decreased (Fig. 4, red trace; Cohen’s *d* = −0.96; Wilcoxon rank-sum test, *p *<* *10^−5^), while that for leftward saccades changed only slightly (*d* = −0.35, *p *=* *0.03). Consequently, the directional modulation disappeared during optical stimulation (*p *=* *0.51). The same optical stimulation slightly reduced spontaneous activity in the blank trials, but these changes were not statistically significant (*d* = −0.54, *p *=* *0.38; Fig. 4*A*, right). Thus, the effects of optical stimulation were specific to the saccade direction, suggesting that the corticothalamic projections may play a role in shaping the direction selectivity of this example neuron.

We also found that the effects of optical stimulation can be temporally specific. Another VL neuron in Figure 4*B* exhibited a slight increase in firing rate after saccades in both directions in trials with no stimulation (black traces), but the activity was greatly enhanced in the presence of optical stimulation (red traces; rightward saccades, *d *=* *1.19; leftward saccades, *d* = 1.71). Interestingly, although neuronal activity before self-timed saccades remained unchanged (Fig. 4*B*, left, middle), the same optical stimulation slightly but significantly suppressed spontaneous activity during the blank trials (*d* = −1.11, Wilcoxon rank-sum test, *p *<* *0.01; Fig. 4*B*, right). Neurons in Figure 4*C* also exhibited saccade-related activity that peaked before saccades, but the effects of optical stimulation were again evident only after saccades (rightward saccades, *d *=* *1.62; leftward saccades, *d* = 1.86). Optical stimulation also elevated the spontaneous neuronal activity (*p *=* *0.02). These results indicate that the signals from the cortex can modulate thalamic neuronal activity in a temporally and spatially specific manner.

However, in many neurons we also observed overall, non-task-specific changes in the firing rates. For example, neurons in Figure 5, *A* and *B*, exhibited a decrease and an increase in firing rates during optical stimulation, respectively, regardless of the behavioral conditions (measured for the entire stimulation period; Fig. 5*A*: rightward saccades, *d* = −1.66; leftward saccades, *d* = −1.61; blank trials, *d* = −1.11; rightward saccades, *p *=* *3.2 × 10^−6^; leftward saccades, *p *=* *7.2 × 10^−6^; blank trials, *p *=* *0.04; Fig. 5*B*: rightward saccades, *d *=* *0.81; leftward saccades, *d *=* *1.37; blank trials, *d *=* *0.70; rightward saccades, *p *= 0.02; leftward saccades, *p *=* *7.8 × 10^−5^; blank trials, *p *= 0.03). Importantly, such non-task-specific changes in activity were also found in monkeys without the vector injections. Figure 5*C* plots such an example recorded from the right thalamus of monkey B (control animal), which exhibited an overall increase in firing rates during optical stimulation, although the effects during blank trials were not statistically significant (rightward saccades, *d *=* *0.92; leftward saccades, 0.94; blank trials, 0.52; rightward saccades, *p *=* *2.0 × 10^−3^; leftward saccades, 2.3 × 10^−3^; blank trials, 0.31, respectively). These changes could have resulted from a thermal effect, as recently reported in rodents (Owen et al., 2019).

### Quantification of task-specific and non-task-specific effects of optical stimulation

To assess the effects of optical stimulation across the population of neurons, we classified individual neurons into four groups according to the presence or absence of task-specific (S) and non-task-specific (N) effects. The non-task-specific modulation for each neuron was evaluated by comparing the spike counts during the entire stimulation period with those during the corresponding period in the randomly interleaved nonstimulation trials. The task-specific modulation was assessed for each of the four time epochs (cue, delay, saccade, and postsaccade periods); the effect size of optical stimulation for the specific timing and saccade direction was compared with the permuted data (see Materials and Methods). Figure 6*A* summarizes the results for the animals with (monkeys F and M, Injection) and without (monkeys B and Q, Control) vector injection. The proportions of the four categories differed between the animal groups (χ^2^ test: χ^2^ = 17.7, *p *<* *10^−3^). Although the proportions of neurons with only non-task-specific modulation (N+S−; Fig. 6*A*, gray bars) were comparable between the groups (Fig. 6*A*: 0.21 vs 0.18, χ^2^ test, χ^2^ = 0.22, *p *=* *0.64), those with task-specific modulation (S+; Fig. 6*A*, red rectangle) were statistically different (Fig. 6*A*: 0.49 vs 0.18; χ^2^ = 12.8, *p *<* *10^−3^). The proportion of neurons with task-specific modulation exceeded the 95th percentile of the permuted data (0.37) for the injected animals but not for the control animals (0.40), indicating that the task-specific modulation was statistically significant only in the injected animals.

Because the data from control animals contained both thalamic and cortical neurons (Materials and Methods), one might argue that the difference in proportions may be because of the inclusion of the latter population. However, even when we performed the same analysis for the control animals with only thalamic neurons (*n *=* *13), we obtained similar results; the proportion of neurons with task-specific modulation (1 of 13) in the control animal was statistically different from that in the injected animals (Fisher's exact test, *p *<* *0.01) and did not exceed the significance level (0.08 vs 0.46). We therefore concluded that the task-specific effects of optical stimulation on the firing of thalamic neurons occurred only in the animals with vector injections. Conversely, the proportions of neurons with only non-task-specific modulation (N+S−) were greater than chance in both groups (the 95th percentiles of the permuted data were 0.05 and 0.07 for the injection and control groups, respectively), indicating that optical stimulation yielded non-task-specific firing modulation even without opsin expression.

A recent study has shown that certain thermal effects of optical stimulation on neuronal activity start as early as several hundred milliseconds (Owen et al., 2019). We speculated that the thermal effects could have longer latency than the opsin-mediated effects, especially for relatively weak optical stimulation, as was used in our experiments. Figure 6, *B* and *D*, plots the time courses of the proportions of neurons with significant stimulation effects (200 ms bins with 50 ms steps) in different conditions (self-timed saccade trials and blank trials) for the animals with (Fig. 6*B*) and without (Fig. 6*D*) vector injection, respectively. When we compared the data with the time courses of the 95th percentiles of permuted data (Fig. 6*B*,*D*, dotted lines), the stimulation effects seemed to start earlier in the vector-injected animals than in the control animals in both conditions. When the latency of stimulation effect was measured with a 10 ms resolution (see Materials and Methods), the values were 230 ms (Task) and 70 ms (ITI) for the population of neurons in the animals with vector injections (Fig. 6*B*, downward arrows) and 890 and 820 ms, respectively, for that in the control animals (Fig. 6*D*). We also measured the latency of the stimulation effects in individual neurons (see Materials and Methods) and confirmed that the latency was shorter in the vector-injected animals than in the control animals (one-sided Wilcoxon rank-sum test, *z *=* *2.44, *p *<* *0.01; Fig. 6*C*).

In addition to the latency of the stimulation effects, we also found that the direction of firing modulation during optical stimulation differed between the two animal groups. The effect size of optical stimulation calculated for all conditions (four epochs and two saccade directions for each neuron) averaged 0.01 ± 0.54 (*n *=* *832 cases; range, −1.65 to 2.09) and 0.16 ± 0.40 (*n *=* *360 cases; range, −0.80 to 2.07) for the animals with and without vector injections, respectively. These values were statistically different (two-tailed *t* test, *t*_(1190)_ = 4.77, *p *<* *10^−5^). When we considered the cases with statistically significant effects (based on the permutation test; see Materials and Methods), the number of cases with facilitatory and suppressive effects were balanced in the vector-injected animals (111 and 111 cases, respectively; Fig. 6*E*, left), but were strongly biased toward a facilitatory effect in the control animals (54 and 7 cases, respectively; Fig. 6*E*, middle left). These proportions were statistically different between the two animal groups (χ^2^ test: χ^2^ = 29.2, *p *<* *10^−7^). This was true even when the data of cortical neurons were excluded from the analysis (Fisher’s exact test, *p *<* *0.01; no thalamic neurons in the control animal showed suppressive effects). In the control animals, the number of cases with facilitatory effects exceeded that of the 95th percentile of the permuted data (54 vs 14), whereas the number of cases with suppressive effects did not (7 vs 15), indicating that the occurrence of facilitatory effects only was significantly greater than chance.

We obtained similar results when stimulation effects were examined during the entire stimulation period. Although the numbers of cases with facilitatory and suppressive effects were comparable in the vector-injected animals (45 and 43 cases, respectively; Fig. 6*E*, middle right), those in the control animals were strongly biased toward facilitation (19 and 2 cases, respectively; Fig. 6*E*, right). This difference in the proportion was statistically significant (χ^2^ test: χ^2^ = 10.8, *p *=* *10^−3^). Again, the number of cases with suppressive effects in the control animals was statistically indistinguishable from chance (the 95th percentile was 3). Thus, the direction of firing rate changes appeared to be different in the two animal groups with and without opsin expression.

To gain further insight into the task-specific effects of optical stimulation, we compared the number of significant cases for each time epoch in vector-injected animals (Fig. 6*F*, left). Overall, the task-specific effects were observed without any strong bias toward a particular time period during the task. There was no statistically significant relationship between the time epoch and the direction of stimulation effects (χ^2^ test: χ^2^ = 6.22, *p *=* *0.10), while the optical stimulation tended to facilitate neuronal activity during the delay period and suppress it during the saccade period. The effect sizes of facilitation (Cohen’s *d*) averaged 0.71 ± 0.10 (SD), 0.85 ± 0.32, 0.73 ± 0.24, and 1.32 ± 0.47 for the cue, delay, saccade, and postsaccade periods, respectively. These values were significantly different (one-way ANOVA: *F*_(3,29)_ = 5.94, *p *<* *0.01) with greater effect sizes during the postsaccade period than during the other periods (Tukey–Kramer test, *p *<* *0.02). The average effect sizes for suppression in the cue, delay, saccade, and postsaccade periods were −0.59 ± 0.07, −0.71 ± 0.43, −0.77 ± 0.24, and −0.72 ± 0.17, respectively, and they were not significantly different (one-way ANOVA: *F*_(3,25)_ = 0.72, *p *=* *0.55). When the effects of optical stimulation between the two saccade directions were compared, we found that the facilitatory effect during the delay period tended to be more frequent for ipsiversive saccades. The proportions of facilitatory effects for contraversive and ipsiversive saccades were 0.67 and 0.43 (cue), 0.60 and 0.82 (delay), 0.29 and 0.33 (saccade), and 0.67 and 0.45 (postsaccade), respectively. Overall, these results indicate diverse contributions of the cortical input to neuronal activities in the oculomotor thalamus.

Finally, we also examined the number of significant cases for each time epoch in the control animals, although the number of neurons with task-specific stimulation effects in these animals did not exceed chance expectations (see above). The task-specific effects tended to be observed during the later task periods (Fig. 6*F*, right), which is consistent with possible thermal effects.

## Discussion

We attempted to optogenetically suppress the corticothalamic terminals while monkeys receiving halorhodopsin-expressing viral vector injections into the SEF were actively engaged in the saccade tasks. Our optical stimulation yielded task-specific changes in neuronal activity in the oculomotor thalamus. These effects were induced directly by local light delivery because the optical stimulation failed to alter any behavioral parameters. We also found that the optical stimulation caused overall, non-task-specific changes in the firing rate, which were also observed in control animals without vector injections. However, the light-induced changes in control animals had longer latencies than those in animals with opsin expression. Furthermore, in control animals, the optical stimulation mostly increased the rate of neuronal firing, and the rate of occurrence of suppressive effects was not statistically significant, while the facilitatory and suppressive effects were equally found in the animals with vector injections.

### Contribution of corticothalamic projections to signal processing within the motor thalamus

In monkeys with halorhodopsin expression at the corticothalamic terminals, the optical stimulation exerted either facilitatory or suppressive effects on thalamic neurons with approximately equal probability. These results may reflect the fact that the primate thalamus contains inhibitory interneurons that receive direct input from the cerebral cortex (Smith et al., 1987; Ilinsky and Kultas-Ilinsky, 1990). Consistent with our present findings, the previous study also demonstrated both the facilitatory and suppressive effects on optical stimulation applied to the corticothalamic terminals expressing excitatory opsins (Galvan et al., 2016). Based on the long latency and complex physiological responses in thalamic neurons, these authors concluded that the corticothalamic projections might play a modulatory rather than “driver” role in the motor thalamus. In the present study, we further extended this hypothesis by elucidating that the signals through the primate corticothalamic projections can induce the task-specific modulation of neuronal activity during goal-directed behavior along with the non-task-specific modification of baseline activity. The modulatory role of cortical inputs has also been supported by the anatomical evidence that the corticothalamic axons form numerous small terminals at distal dendrites in the motor thalamus (Kuroda et al., 1998; Kuramoto et al., 2011; Rovó et al., 2012).

The motor thalamus relays signals from the basal ganglia, the cerebellum, and the brainstem to the cerebral cortex. Our results suggest that the motor thalamus may not merely transmit an accurate copy of subcortical signals, but may also be actively involved in information processing by integrating signals from the cortex during goal-directed behavior. Thus, the motor thalamus could serve as a unique, unexplored center for achieving neural computations, where cortical and subcortical signals interact before reaching the cortex (Bosch-Bouju et al., 2013). For example, while a specific region in the oculomotor thalamus has been implicated in the monitoring of eye movements by mediating signals from the brainstem (Schlag and Schlag-Rey, 2002; Sommer and Wurtz, 2002), several studies have shown that eye position signals in this region are significantly modified during movements (Schlag-Rey and Schlag, 1984; Wyder et al., 2003; Tanaka, 2007a), possibly because of the unique computation within the thalamus. Thus, the corticothalamic projections themselves might convey highly context-dependent information, or alternatively, the modulatory signals from the cortex might amplify specific subcortical inputs within the motor thalamus under different conditions (Bosch-Bouju et al., 2013; Groh et al., 2014; Yamawaki and Shepherd, 2015).

Our observation of saccade direction-specific effects of optical stimulation suggests that directional information in the SEF might be transmitted to the oculomotor thalamus. These signals could be particularly important for goal-based or object-centered saccade computations in the thalamocortical network involving the SEF (Schlag and Schlag-Rey, 1987, 1990; Olson and Gettner, 1995; Fig. 1*C*). Furthermore, since the manipulation of neuronal activity in the SEF or the thalamus alters the timing of self-initiated saccades (Tanaka, 2006; Kunimatsu and Tanaka, 2012), one of the potential roles of the network may be to adjust saccade timing based on a particular context, which has been well studied in the SEF (Isoda and Tanji, 2002; Berdyyeva and Olson, 2011).

In the present study, the effects of optical stimulation were generally small. Considering the technical difficulty of efficient terminal inhibition (Mahn et al., 2016), we were unable to estimate the impact of the corticothalamic projections on the task-specific modulation of thalamic neuronal activity. Indeed, a recent study reported in rodents that the effects of the optogenetic inhibition of corticostriatal terminals on the firing of striatal neurons were moderate (Lee et al., 2019), although such cortical inputs have been well established to provide strong driving signals to the striatum (Obeso et al., 2008).

The corticothalamic projections originating from layers V and VI are thought to play different roles. In the sensory system, the projections from layer V provide strong driving input while those from layer VI may modify neuronal sensitivity in the thalamus (Sherman, 2016). Recent studies suggest that similar differential roles of cortical inputs from the two layers may also hold true in the motor thalamus (Kakei et al., 2001; Rovó et al., 2012; Bosch-Bouju et al., 2013; Collins et al., 2018). Although the present study was unable to distinguish signals from different cortical layers, it is important to understand how different corticothalamic projections modulate task-related thalamic neuronal activity in behaving animals. Given that layer V corticothalamic neurons overlap pyramidal tract neurons, whereas the layer VI neurons do not (Kita and Kita, 2012; Harris and Shepherd, 2015), retrograde transfection of opsins or chemoreceptors (Tervo et al., 2016; Kobayashi et al., 2017) might be useful to differentiate their roles in thalamic neuronal activity during goal-directed behavior.

### Non-task-specific effects of optical stimulation in the absence of opsin expression

Non-task-specific changes in neuronal firing were observed during optical stimulation in both animal groups with and without opsin expression. In the control animals without opsin expression, the changes in neuronal activity started as early as 820 ms (Fig. 6*D*), and the optical stimulation exerted facilitatory effects in most cases. Such a stimulation effect in the control animals was consistent with recent data in rodents suggesting that optical stimulation might alter neuronal activity by heating nearby tissues (Stujenske et al., 2015; Owen et al., 2019).

Nevertheless, our findings of non-task-specific stimulation effects were rather surprising, because the laser power used in the present study (<5 mW in most experiments) was weaker than those used in previous studies with inhibitory opsins (Camporeze et al., 2018). The duration of optical stimulation in our study (2500 ms) was also shorter than those in many previous studies using optogenetic tools (Wiegert et al., 2017). One reason behind our observations is that data obtained from single unit recordings should be much more sensitive to local temperature changes than those from behavioral analyses, and that most of the previous studies aimed to manipulate the behavior of animals. Another reason might be that we used relatively thin optical fibers (65–125 μm) in this study; thinner optical fibers produce a higher light density that may have greater local thermal effects (Stujenske et al., 2015; Arias-Gil et al., 2016).

Careful confirmation of optimal stimulation parameters is of particular importance in primate brain research. Studies in nonhuman primates may focus on changing neuronal activity rather than behavioral performance, as behavioral manipulation by optogenetics is generally difficult in large animals. In addition, researchers using primates are eager to use thinner fibers to minimize tissue damage for repeated penetrations, which might pose a further risk of tissue heating. As suggested in previous studies (Stujenske et al., 2015; Owen et al., 2019), limiting the stimulation duration and/or the use of pulse trains might be helpful, although the efficacy of stimulation pulses for inhibitory opsins remains uncertain. Especially, in an experimental setup like ours, focusing on a particular time frame using a light duration of <500 ms, for example, might be beneficial in preventing nonspecific stimulation effects, given the latency data in Figure 6, *C* and *D*. Thus, our results underscore the importance of control experiments with optical stimulation in animals without opsin expression. Hopefully, the present work will be useful for many future studies using similar techniques.

## Synthesis

Reviewing Editor: Satu Palva, University of Helsinki Decisions are customarily a result of the Reviewing Editor and the peer reviewers coming together and discussing their recommendations until a consensus is reached. When revisions are invited, a fact-based synthesis statement explaining their decision and outlining what is needed to prepare a revision will be listed below. The following reviewer(s) agreed to reveal their identity: Kiyoshi Kurata.

In this manuscript the authors describe results from manipulation of activity in the motor thalamus through optogenetic suppression of corticothalamic terminals in non-human primates during preparation and execution of saccades. The study was intended to examine roles of direct corticothalamic projections in neuronal activity of ‘motor thalamus’. In the task, monkeys were required to withhold saccades for 1000 to 1700 ms following briefly presented (100 ms) visuospatial target cues. During the period, activity of axon terminals from supplementary eye field (SEF) was continuously inhibited by optogenetic stimulus using halorhodopsin with an optrode, and activity of thalamic neurons in the projection area from the SEF was simultaneously recorded by the optrode. The authors do not find a behavioral effect following optogenetic suppression of corticothalamic terminals but show clear task-related modulation of thalamic activity that is expressed either as facilitation or suppression of activity in the various task epochs. These results highlight a possible modulatory role of corticothalamic projection on thalamic activity. Moreover, they show non-specific mainly facilitatory effects in the control (non-injected) animals during optical stimulation, a fact that indicates that thermal effects may induce non-task-specific facilitation, which should be taken into account by performing appropriate controls. Given that optogenetics in primates still present significant challenges, the methodological details and the results presented here can provide useful insights to future studies both for designing and executing targeted manipulations of activity in non-human primates with optogenetics.

Major comments

1) It is stated in Introduction that they examined whether optogenetic suppression of corticothalamic terminals could induce task-specific neuronal modulation in the motor thalamus when the animals performed saccade tasks (p.4 lines 6-7). Because some prominent aspects of the projection have been revealed (e.g. Olson et al. 2012), this description seems too general, and it is not clear why the authors specifically selected (1) the SEF for the stimulation site, and (2) halorhodopsin to inhibit corticothalamic projection specifally in the self-timed oculomotor task (p.6 lines 1-18), although it is briefly described in the result section of the manuscript (p.11 lines 14-15, no references are cited). It is expected that inhibition by halorhodopsin on activity of thalamic neurons could be readily effective if corticothalamic neurons exhibit high frequency discharge, as in globus pallidus. Otherwise, it is expected that effects of halorhodopsin may not be evident in behavioral performance which induces vigorous activation of the corticothalamic neurons. Importantly, it was reported that muscimol injection in thalamus induced behavioral deficits in performing the same task as in this study (Tanaka 2006). Thus, it can be assumed that the authors tried to find a role of selective inputs from the SEF of monkeys performing the same task by examining behavioral deficits induced by optogenetic inhibition with halorhodopsin. The authors are encouraged to state their rationale for this study more clearly.

2) It is stated in Methods that optogenetic stimulations were delivered for 1700, 2000, or 2500 ms covering the self-timed period following the brief target presentation. However, it is not clearly stated in Methods that the stimulation was continuously applied in step-like manner, not using pulse trains, although it is implied in Discussion (p.22 lines 8-10).

3) In Results, histological images are presented in Figure 1 to show recording sites where received projections from the SEF, revealed by expression of EYFP with halorhodopsin. It is stated in the text that the recording site was located within the centrolateral nucleus of the thalamus (CL) and adjacent regions (Figure 1G) in which axon terminals were observed in a thin strip form (Figure 1F). The CL is generally regarded as nonspecific nucleus, because it receives abundant inputs from brain parts in addition to those from basal ganglia and cerebellum (Figure 1). In my knowledge, the main body of the motor thalamus receiving projection from the SEF is more rostrolateral parts of the motor thalamus such as VA, nucleus X, VLo, and VLc (Huerta & Kaas 1995, Bodor et al 2008). Thus, I recommend the authors to show another figure indicating neuronal locations within the thalamic nuclei in more rostral histological sections.

4) Inhibition of activity in axon terminal following optogenetic stimulation is not directly shown in Results. Because optrodes were used in this study, I recommend the authors to exhibit that neuronal activity within the SEF injection site was suppressed in response to optogenetic stimulation, as Galvan et al (2016) showed that neurons in the primary motor cortex of monkeys were activated shortly after optogenetic stimulation using channel rhodopsin 2 (ChR2) or C1V1. Galvan et al. (2016) also showed that stimulation of corticothalamic efferents resulted in relatively long latency excitatory and inhibitory effects on the thalamic neurons they recorded. The results should be described in Discussion to compare effects of corticothalamic projections driven by ChR2 with those using halorhodopsin in this study.

5) In Discussion, it is stated that the modulation by corticothalamic projection could play roles in goal-directed behavior. But this description seems too general. Because the SEF is involved in goal-directed saccade, as shown in Figure 1C, as opposed to the frontal eye field that controls constant vector saccades. It is discussed in the text that the corticothalamic projection plays a role in monitoring eye movements, including eye position during saccades (p.20 lines 3-8). However, it is also shown in this manuscript that some neurons exhibited activation preceding saccade onset (Figure 3). This observation corresponds to the evidence that the thalamic nuclei receive inputs from superior colliculus, and suggests that the thalamic neurons are also involved in computation of goal-directed saccades (Schlag and Schlag-Rey 1987) or saccade in object-centered coordinate (Olson and Gettner 1995). Thus, it should be discussed what role the neuronal modulation by the corticothalamic projection from the SEF could play in goal-directed saccades, regardless of whether the projection acts as ‘driver’ or ‘modulator’ (Sherman and Guillery, 1998; Rovó et al. 2012).

Specific comments - minor

1) There is no mention on the depth of injection. Information from this or previous studies regarding the likely spread in all three dimensions would help better appreciate the area of infection.

2) Although the authors mention that recording locations were reconstructed in based on electrode tracks and electrolytic lesions the only figure that they provide does not help in assessing the extent and location of recordings.

3) In Figure 1F it is hard to appreciate which part(s) of the thalamic nuclei are labeled. A Nissl stained neighboring section showing the different nuclei or white borders of the thalamus and the different nuclei on the phot of Fig 1F would help.

4) In lines 276-278 the authors report "...that our optical stimulation modulated only small number of neurons and did not affect behavioral performance, indicating that any change in neuronal activity during optical stimulation resulted from the direct stimulation effects but not from behavioral changes". This is not clear at all. Why would neuronal activity changes be the result of behavior rather than behavior be the result of changes in neuronal activity? Moreover, given that at this stage in the manuscript no results on changes in neuronal activity have been presented it sounds even more odd to make this claim. I would suggest that they end the sentence at "behavioral performance.

5) In the example neurons shown in Figure 4 no statistics are given for the described changes.

6) Lines 496-497. "...focusing on a particular time frame using a light duration of < 500 ms might be beneficial in preventing nonspecific stimulation effects (Fig. 5D)". It is unfortunate that the authors have not tried different stimulation parameters that would help elucidate how nonspecific effects could be mitigated. Be that as it may, it is not clear to me how the reference to Figure 5D relates to the statement.

5) p.3 line 4 from the bottom: the word, ‘polarity’, is ambiguous. I recommend to rephrase it.

6) p.3 line 9: the reference, Asanuma et al. (1983 in Brain Research), is not appropriate here. I recommend to cite other references, such as Asanuma et al. (1983) in Brain Research Reviews.

7) p.6 line 9: the remembered cue location, instead of the cue location.

8) p.7 lines10-12: I recommend to describe intensity of the laser light in mW/mm2, instead of mW, to allow comparisons with other optogenetic studies and to discuss its thermal effects described in the manuscript.

9) Some references do not follow the journal’s format on capitalization of reference title.
